# Use of the data system for field management of a clinical study conducted in Kolkata, India

**DOI:** 10.1186/s13104-015-1767-7

**Published:** 2016-01-09

**Authors:** Ju Yeon Park, Deok Ryun Kim, Bisakha Haldar, Aiyel Haque Mallick, Soon Ae Kim, Ayan Dey, Ranjan Kumar Nandy, Dilip Kumar Paul, Saugata Choudhury, Shushama Sahoo, Thomas F. Wierzba, Dipika Sur, Suman Kanungo, Mohammad Ali, Byomkesh Manna

**Affiliations:** International Vaccine Institute, Seoul, South Korea; National Institute of Cholera and Enteric Diseases, Kolkata, India; B.C. Roy Post Graduate Institute of Pediatric Sciences, Kolkata, India; PATH, Washington, DC USA; Johns Hopkins Bloomberg School of Public Health, Baltimore, USA

**Keywords:** Data management, Field management, Database, Data system, Clinical study

## Abstract

**Background:**

Designing an appropriate data system is important to the success of a clinical study. However, little information is available on this topic. We share our experiences on designing, developing, and implementation of a data system for management of data and field activities of a complex clinical study.

**Methods:**

The data system was implemented aiming at determining the biological basis for the underperformance of oral vaccines, such as polio and rotavirus vaccines in children at a site in Kolkata, India. The system included several functionalities to control data and field activities. It was restricted to authorized users based on their access privileges. A relational database platform was chosen, and Microsoft Visual FoxPro 7.0 (Microsoft Corporation, Seattle, WA, USA) was used to develop the system. The system was installed at the clinic and data office to facilitate both the field and data management activities.

**Results:**

Data were doubly entered by two different data operators to identify keypunching errors in the data. Outliers, duplication, inconsistencies, missing entries, and linkage were also checked. Every modification and users log-in/log-out information was auto-recorded in an audit trail. The system offered tools for preparation of visit schedule of the participants. A visit considered as protocol deviation was documented by the system. The system alerted field staff to every upcoming visit date to organize the field activities and to inform participants which day to come. The system also produced a growth chart for evaluating nutritional status and referring the child to a specialized clinic if found to be severely malnourished.

**Conclusion:**

The data system offered unique features for controlling for both data and field activities, which led to minimize drop-out rates as well as protocol deviations. Such system is warranted for a successful clinical study.

## Background

Clinical studies play important role for improving human health; therefore, these studies should be managed properly and carefully [1, 2]. Data collection, data cleaning, editing, and management of the data in compliance with regulatory standard and the International Conference on Harmonization Harmonized Tripartite Guideline for Good Clinical Practice (ICH GCP) are important aspects of a clinical study. A data system should ensure that these issues are well addressed in the system design [3]. The design should also ensure that the data are accurate, complete and compliant with regulatory standards and GCP requirements, and the analyses are done using cleaned data sets. Besides, the system should include tools for providing support to the clinic, field and laboratory staff in order to collect data accurately and in a timely manner. Only a few trials goes exactly as initially planned. For instance, a case report form (CRF) may need updating in the course of trial, a new trial site may be added, and new technology may emerge [4]. An investigator is, therefore, to worry about installing a data management system that is flexible and compliance with set guidelines and standards.

Although literature is plentiful in describing various aspects of data analysis, little can be found that tells about practical aspects of designing a data system [5–8]. Rarely, a data system includes tools to perform both data and field activities. It is worth mentioning, analyses of the clinical studies can be flawed not only by problems in data acquisition and field methodology, but also by errors in the design of the data system.

Recent hardware and advanced software tools have made it possible to develop an ideal data system for clinical studies [5]. In designing the system, one should consider study objectives, nature of the study site, and the local issues. The design should be flexible to accommodate unanticipated issues in the field and data management. Inclusions of tools for checking data outliers and inconsistencies as well as for queries are essential. Storing other information such as metadata (data dictionary) and audit trail, that tracks all the modifications ever made in the system, must be included in the system. While designing, one should also focus on system specifications, structure of the database, data and field management tools, and performance of the system.

We designed, developed, and implemented a data system for a complex clinical study in Kolkata, India. In the study, the vaccines was given to the participants concomitantly with the EPI routine immunization program for a period of 1 year, and there were multiple visits for specimen collection from the study participants, which could have been well managed with the support from the data system. This paper discusses the design, development, and implementation, as well the performance of the data system.

## Methods

### The clinical study

The clinical study named provide (Performance of Rotavirus and Oral Polio Vaccine in Developing Countries) was initiated in Kolkata, India with the hypothesis that there are factors for the underperformance of oral vaccines in children living in the developing world. It aimed to determine whether decreased vaccine responsiveness to oral poliovirus or rotavirus vaccines is associated with the presence of tropical enteropathy (TE); and to evaluate whether the impact of an inactivated polio vaccine (IPV) boosted systemic and mucosal immune responses to polio vaccines following vaccination with oral polio vaccine (OPV) in children with and without TE. The study was carried out among infants residing in Kolkata. The study aim was to recruit 372 children at 6 weeks of age and follow them until they were 53–54 weeks of age. There were 12 visits for each of the participants during the follow-up time including the initial visit. This study was also carried out in accordance with the routine immunization schedule in India. Therefore the participants would have to be vaccinated with other vaccines, such as, BCG, DPT, HepB and Measles.

### The data system

A custom-made data system, named INDSys, was designed and developed aiming to turn the information from the participants into data, to transcribe the data into a database without errors, and to manage data delivery from clinics and laboratory efficiently. The design phase focused on defining the database components and the modules and interfaces required for satisfying the need of data and field activities. While developing the system, provisions were kept for accommodating any new data or field related issues into the system. The system was developed using Microsoft Visual FoxPro 7.0 (Microsoft Corporation, Seattle, WA, USA), and the data was managed in a relational database environment. All related data, including clinical, laboratory and other data sets as well as field work schedule, were integrated within the data system.

The data system was restricted to authorized users only such as data managers, data operators, clinical monitors and investigators. There were two levels of restrictions in the system. At the first level, the users required log-in identification and password to access the system. At the 2nd level, the system functionalities were restricted according to the user’s privileges. The functionalities according to the user’s role are shown in Table 1. The data entry screens were designed to look similar to the data forms for facilitating the data entry operations. A dual data entry system was designed to avoid keypunching errors in the data [9]. A comprehensive data validation tool was incorporated in order to identify missing values, outliers, duplications, and intra- and inter-record inconsistencies in the data. Errors that cannot be corrected, such as unordinary data or missing laboratory results in the system, were also selected to remain in the database with proper documentation. The data system automatically stored information on changes in the data, users’ log-in, log-out, entry, modification, and deletion of the data in an audit trail. Information such as who changed the data and when the changes were made were also stored in the audit trail. Additional information such as a data dictionary, data status, data checking plan and code plans for open-ended questions were accessible through the system. A report generation module was included in the system to monitor the performance of field and data activities.

**Table 1:** Accessible functionalities according to the user’s role

Role	Main responsibilities	Accessible functionalities
Database administrator	Grant permission to a user for accessing system and maintain system and troubleshooting	All functions
Data manager	Supervise data entry, error check, and backup	Entry, view/edit/delete, dual check, error check, report/query, maintenance(backup, change password)
Data operators	Data entry and check dual entry error	Entry, view/edit, dual check, maintenance (change password)
Clinical monitor(s)	View data for monitoring electronic data	View, report/query, maintenance (change password)
Investigator(s)	Supervise all data activities	View, report/query, maintenance (change password)

For the field management, the system design included activities in field office, such as participant’s contact, calendar of all visits, participant’s discontinuation/drop-outs, growth chart, missing visits, etc. Necessary tools (*vide infra*) were included in the system so that the field management could control the activities easily and smoothly, especially for this complicated schedule of visits. Each participant was to be vaccinated with several vaccines (e.g. DPT, HepB, and Oral Polio Vaccine) in accordance with Expanded Program on Immunization (EPI) in India, and study vaccines (e.g., Rotavirus, Oral Polio Vaccine, or Inactivated Polio Vaccine). If a participant received a vaccine on a day other than the scheduled day, the subsequent date of vaccination as well as scheduled dates of visit for specimen collection would also change to the date of vaccination and a new calendar for the visitation schedule was prepared for the participant accordingly. The data system included tools to generate the new visitation schedule after each visit was made by a participant.

### Implementation of the data system

The data system was implemented in standalone computers at the data center and at the field clinic of the National Institute for Cholera and Enteric Disease (NICED). Microsoft Windows was chosen as the operating system to implement the data system. No other software was required to operate the system. The data and field staff of the project were trained on how to operate the data system. Authorized users, had access to the system through assigned identification and password. The staff members at the clinic had access to the system only for management of the field activities. The database at the clinic was continuously updated by the data staff of the project to facilitate field activities with updated data sets.

### Ethical considerations

Written informed consent was obtained by mothers of participating children. The study protocol was approved by the scientific advisory committee (SAC), Institutional Ethics Committee of the National Institute of Cholera and Enteric Diseases (NICED), the Health Ministry Screening Committee of India, and the International Vaccine Institute Institutional Review Board. The protocol was registered at clinical trial registry of India (CTRI/2012/03/002504) and at clinicaltrials.gov (NCT01571505).

## Results

### Start-up screen

**Fig. 1:**
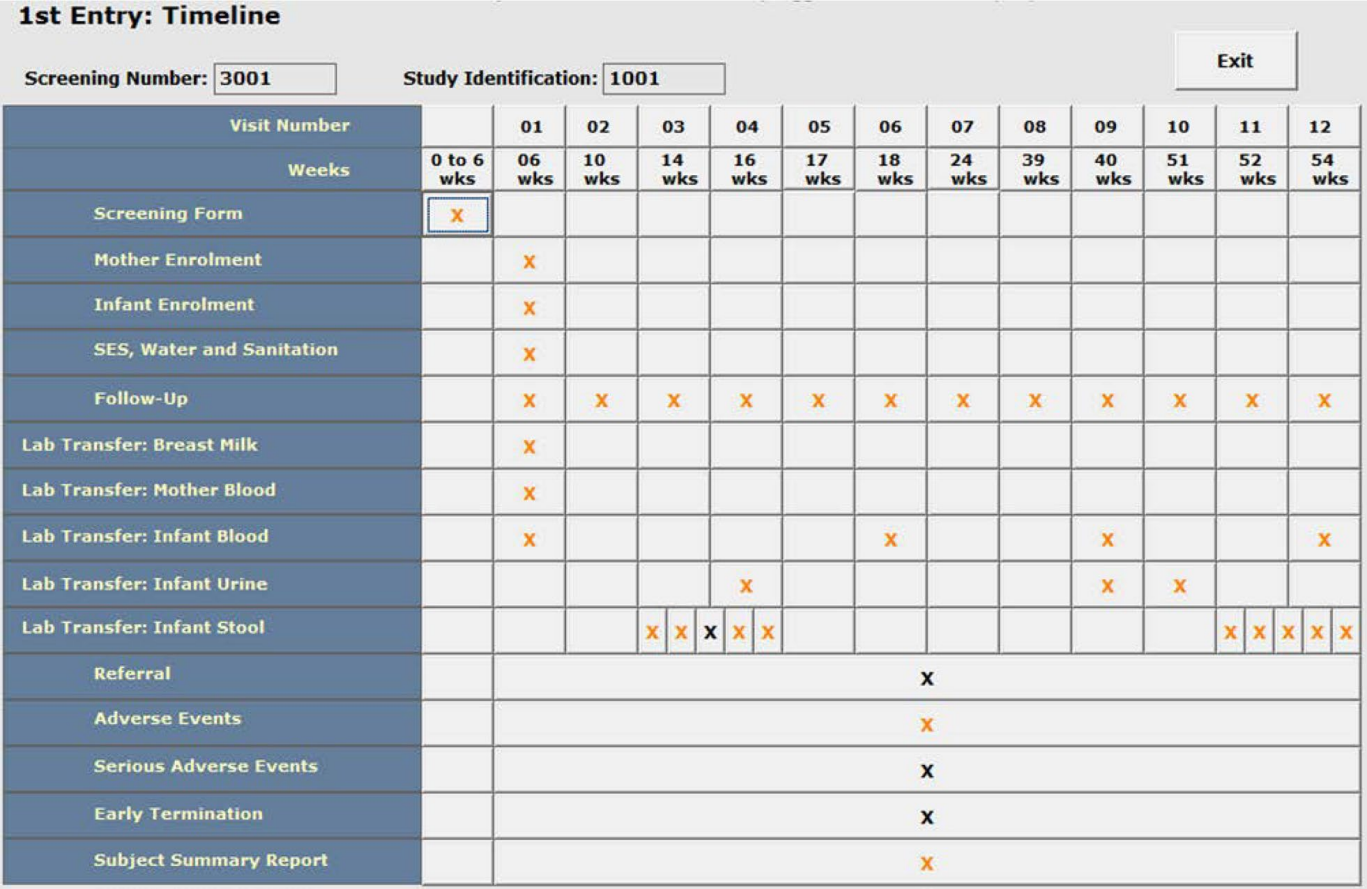
The status of data entries of the different forms of a participant (start-up screen)

Upon starting the system, a log-in screen appears for accessing the system. After successful log-in, a menu describing the list of activities appears on the screen according to the user’s privileges. As a dual data entry system, the system provides options for entering the data either in the 1st or 2nd file according to the user’s privileges. If the operator enters a Screening Number for a new recruitment, the system shows the Data Entry Status screen (called start-up screen) of the participant (Fig. 1). In this screen, the data entry status for the different visits of the participant is reflected by color, and the number of successful and missed visits are easily identifiable. Since the screening form is used to assign the Study ID, the user will receive an error message if s/he tries to enter any other forms before entering the screening form. In the event of early termination of a participant, the operators are not allowed to enter any other forms of the participant. When one-year of follow-up of a participant is passed, the system checks for missing visits and specimens, and alerts accordingly. This helps ensure completeness of the data entries of all visits of a participant.

### Data entry and editing

The study had two sources of data: clinic and lab. The data from the clinic, such as enrolment, follow-up, and specimen collection were entered into the data system soon after receiving the forms. The lab data were entered and verified by lab scientists following data validation rules and then uploaded onto the system. The embedded data validation process in the system ensures quality of the data after checking for outliers and inconsistencies.

While entering the data from the clinic, the other functions are inactivated by the system. In the event of a potential error during data entry, the system warns the operator and provides options for correcting it. However, the operator can move on without correcting the data if the correction cannot be made at his/her level. A specific module for data checking is incorporated into the system (vide supra) to trap any error in the database.

The system provides a view screen for reviewing all data forms in one screen. The user can view the data by using navigation keys located on the right side of the page of the screen and selecting the participant ID, or by entering the participant ID in a specified box. If a participant ID is selected, the participant’s data form can be viewed by clicking on a specific tab of the form abbreviations.

The lab data, before final submission to data room, were recorded on specific data forms designed for lab results, dually entered in Excel, and further checked by lab personals. The data generated by the laboratory is uploaded onto the system from the soft copy of the data. In this case, the user clicks onto the Upload Lab Result tool to get the list of the file names of all the lab data. If a wrong file name is selected, the system warns the user and prevents him/her from uploading the data.

Unlike clinic data, the lab data are not allowed to edit directly on the screen. If an error is detected in the lab data, the error is sent to the laboratory for correction. After getting the corrected data in a soft copy, the erroneous data was replaced with the corrected one.

### Data validation and audit trail

There were two modules in the system for checking accuracy of the data. Firstly, the data are doubly entered to detect keypunching errors. The system provides a list of discrepancies between the two entries including unmatched unique identification of the children between the two entries. Once the keypunching errors in the data are resolved, the user is allowed to move to the second step of data checking which includes outliers, inconsistencies, validity of the dates, and linkage. If the errors generated at the source, the forms along with the type of errors are sent to the field office for resolution. Once the error feedbacks are received, the data are updated accordingly. When an update is done, the user ID, date and time of update, and old and new values are stored in the audit trail. Audit trails are being created incrementally, in chronological order, and in a manner that it does not allow new audit trail information to overwrite existing data. If updates are made multiple times, the users can trace complete history of the updates.

### Producing schedule of visits

**Fig. 2:**
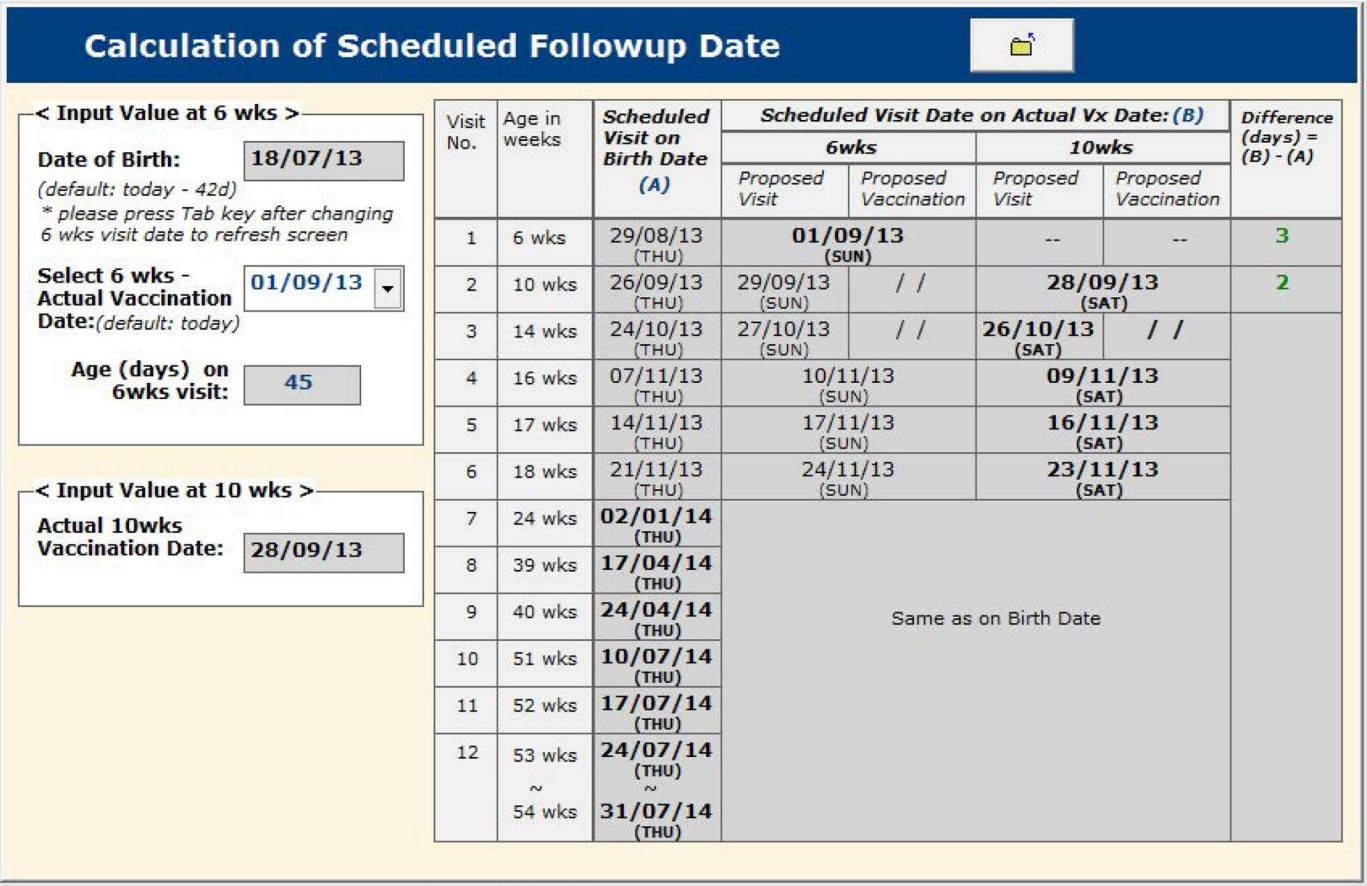
The schedule of visit dates at the clinic of a participant

**Fig. 3:**
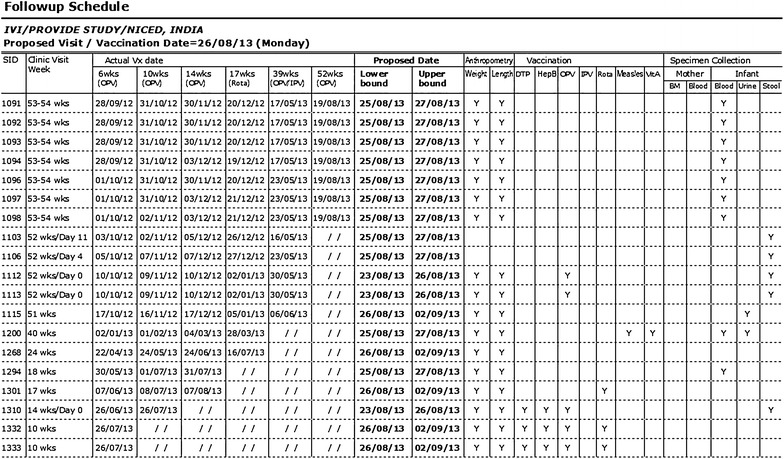
List of the participants to be visited on the upcoming clinic day

As mentioned above, there were 12 visits for each participant in a year including the initial visit for consent and screening of the participants. It was necessary to calculate the scheduled dates of visit for each participant during the follow-up period, and remind them to come to the clinic on that date. Since enrolment dates differed from one participant to another and the purpose of visit also differed from one visit to another, keeping track of the schedule dates and purpose of visit would be difficult without having field management tools in the system. Note that the scheduled dates of visit might also change depending on the last date of visit. The system produces a fresh calendar of the scheduled dates of appointments after every visit is made (Fig. 2). This helped Field Supervisors to fill the dates of visit schedule in the Study Identification Card which should be brought when the participants come to the clinic, to remind them by telephone and home visit prior to the schedule date, and to identify whether the visits were made on the planned date or not. The system also produces the list of all visits to be made on the upcoming clinic day (an example is shown in Fig. 3), so that the field staff can take necessary actions to make those visits successful.

### Data backup

Data can be lost by a computer accident or catastrophic loss -such as losing a critical file or experiencing unexplained file corruption, a hard drive crash, or total system loss. The system provides a backup tool to maintain a regular backup at the end of each working day. A backup copy was kept in a separate building away from the data center.

### Reporting protocol deviation

One of the most challenging tasks of the clinical study was to manage the scheduled dates of visits of the participants for vaccination and specimen collection. The target visit dates may change depending on the previous date of visit of the participants. For visit week 6–18, participants will be invited to come to the clinic based on the date of his/her last vaccination on account of the mandatory 4 weeks window between vaccinations. For visit week 24–53, participants will be at age of the visit week +7 days, regardless of the date of last vaccination. If a visit or procedure cannot be made within the specific time period (called window period), then those visits or procedures are treated as protocol deviation. Since this clinical study depends largely on the biological issues, any protocol deviation would have negative impact on the results of the analysis. Therefore, it was important to keep the number of protocol deviations as minimized as possible.

**Fig. 4:**
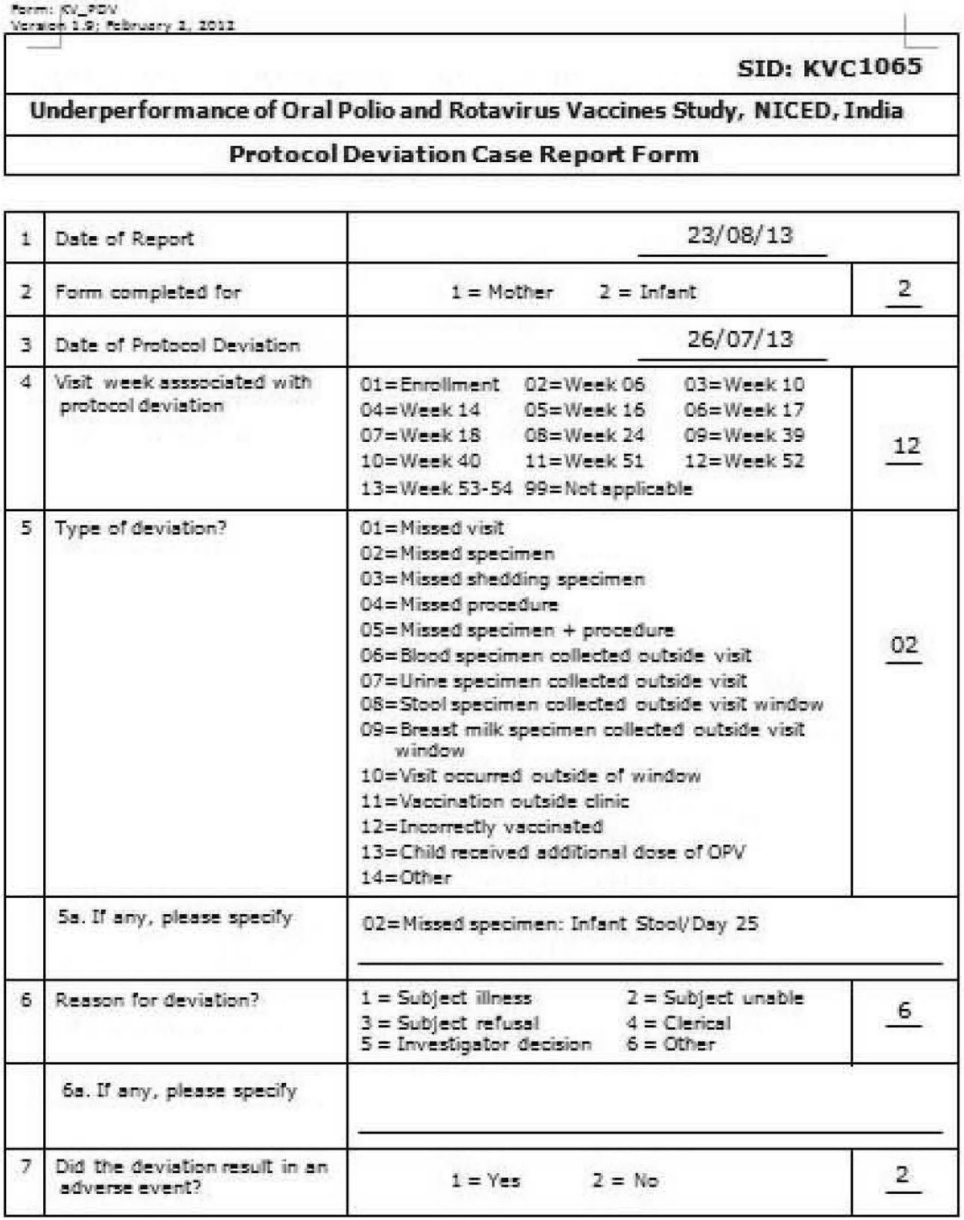
A protocol deviation form generated from the data system

**Fig. 5:**
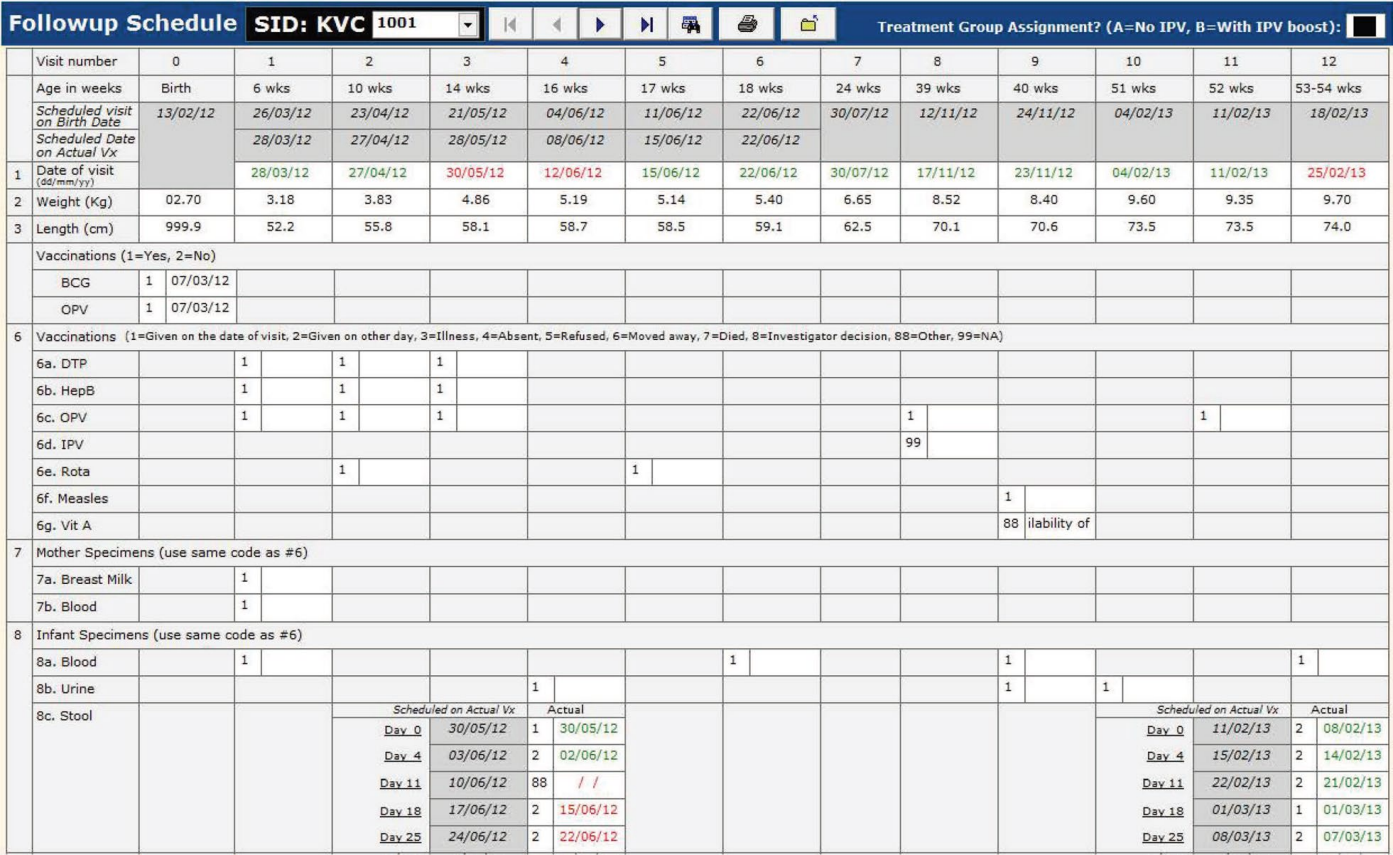
The status of visits and vaccinations, and sample collections with protocol deviation status generated by the data system

The data system produces how many visits or procedures turned out to be protocol deviation. Note that the system generated protocol deviation is data driven, thus the study could overcome limitation of manually created protocol deviation form, which is prone to error and may differ from the data actually stored in the database. An example of the system generated protocol deviation is shown in Fig. 4. The system also provides the status of all visits, vaccinations, and sample collections with (in red color) and without (in green color) protocol deviation (Fig. 5).

### Reporting of severely malnourished children

**Fig. 6:**
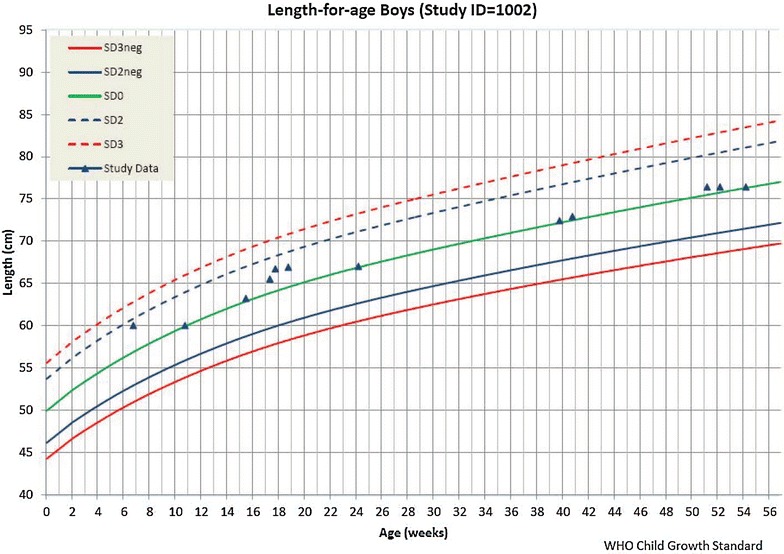
A growth chart generated by the data system

**Fig. 7:**
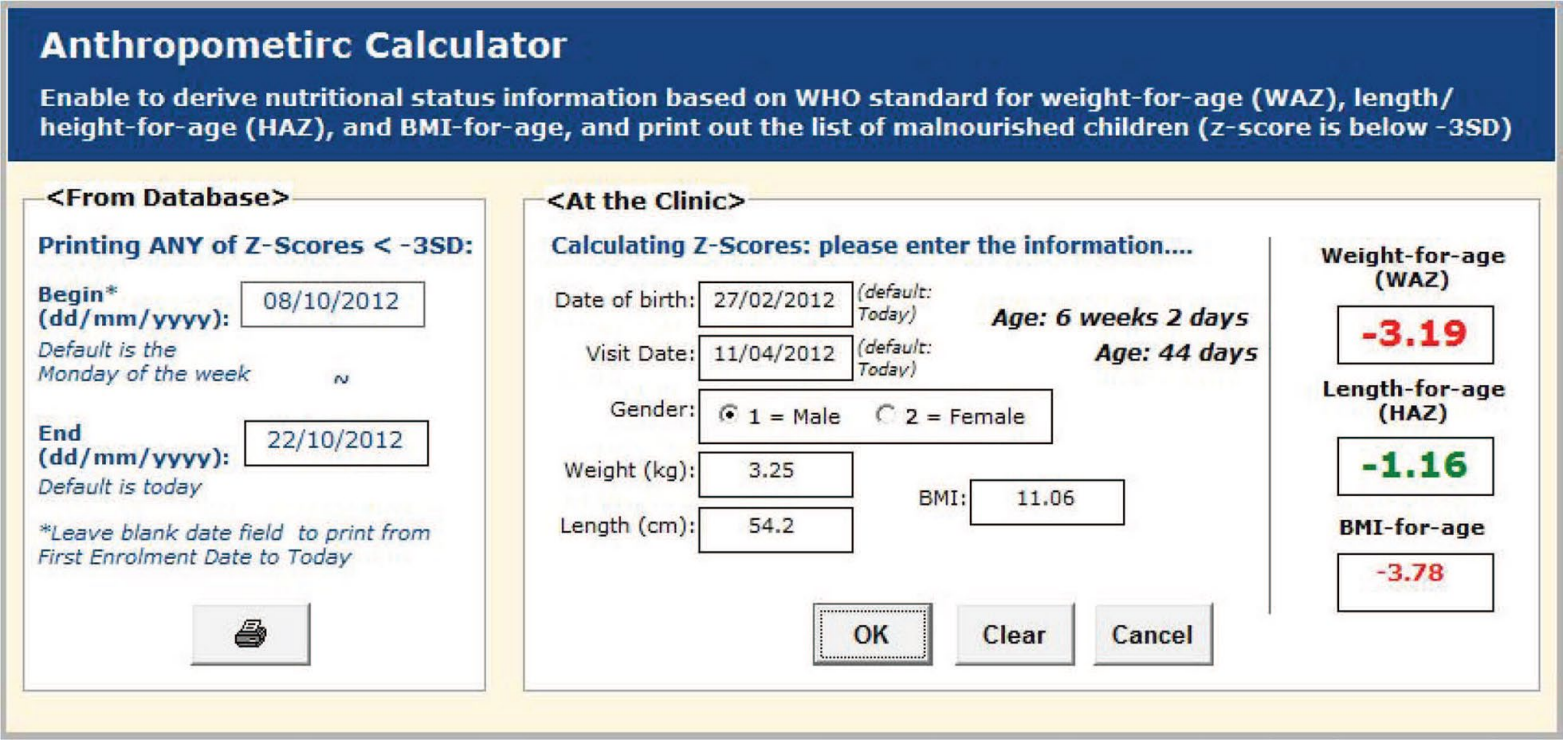
An anthropometric calculator

**Fig. 8:**
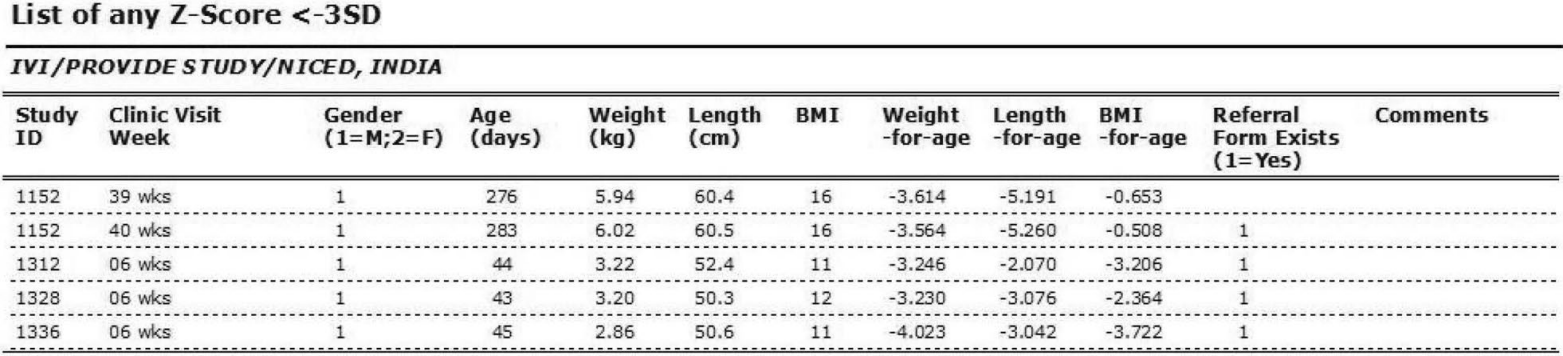
The list of participants malnourished children (z-score is below -3SD) generated by the system

The study required referring severely malnourished children to a specialized clinic for treatment. Upon request, the system provides an anthropometric data calculator for calculating nutritional status and drawing growth charts of the children, which were evaluated to understand the nutritional status of the children. The system produces two types of growth chart for each participant: weight-for-age and length-for-age. By triggering the participant’s ID, the user can instantly get the child’s growth status according to the World Health Organization (WHO) child growth standard (http://www.who.int/childgrowth/standards), as shown in Fig. 6. In the figure, the two lines from the bottom were related to malnutrition status. If the child’s weight or height is marked below the line of SD2neg (<-2SD), the child is considered malnourished. If the child’s weight or height is marked between line of SD2neg and SD3neg, the child is considered moderate malnourished. And, if the child’s weight or height is marked below SD3neg (<-3SD), the child is considered severely malnourished. In case of severely malnourished, the child was referred to a specialized clinic for treatment. However, with this growth chart, the user could not get z-scores for anthropometric indicators (weight-for-age and height-for-age) based on the WHO child growth standard. Thus, the list of severely malnourished children generated from the system was just to facilitate field activities (Figs. 7, 8).

## Discussion

Running a clinical study smoothly like ours is challenging due to its complex and long duration visit schedule. In our study, all 372 participants were successfully recruited according to the planned schedule. The drop-out rates were only 8 % and protocol deviations stood at 3 %. The reasons for these protocol deviations included: visits occurring outside the window period (44 %), missed specimen (34 %), missed visits (11 %), specimen collected outside the window period (10 %), and missed procedures (1 %). 99 % blood samples, 95 % stool samples, 98 % urine samples, and 100 % breast milk samples were successfully collected. Such an excellent performance would have not been possible without having the support provided by the data system.

In addition to manage data activities and generate data reports, our data system provides support for management of the field activities such as preparing visit schedule, alerting next day visit schedule and assessing requirement of the logistics including number of doses of the vaccines, etc. for the next visit schedule. It also ensures a good clinical practice, a legal requirement to conduct clinical trials in many countries. The other advantage in our system is that it generates nutritional status report of the participants at each time point so as to take immediate actions against severely malnourished infants, such as referring to a specialized clinic where proper treatment can be ensured. In the system, the data interoperabilities are limited to Acrobat Reader and Excel, as required by us. However, we have kept provision for the data interoperability in any other standard data format.

Our data system is specific to our study, which is unlikely to match exactly with any other studies. Three-month time of a skilled system designer and programmer was required to design and develop the system. Unlike open source software where many developers have the opportunity to scrutinize the system, our system was scrutinized in-house.

Since many developers scrutinized the open source application, it is hard for bugs to hide in that application. Also, since codes of the open source software are shared among numerous parties, it is typically well structured, which cannot be ensured a single company develops like us [10].

However, our system passes through different courses of action over the time, and we were able to fix the bugs in the system. More importantly, we were able to tailor it specific to the project activities, which may not have been possible in an open source software solution.

The limitation in our system is that it was installed on standalone computers. Therefore, we had to upload of the updated database to the field office computer on a regular basis. Similarly, the data generated at the lab were uploaded manually into the system. This offline mechanism could have been avoided if the computers at the clinic and lab were connected to the data office through a network. However, we could not make it possible in our setting due to limitation of resources.

Still a large part of clinical centers uses their own developed solution or a single solution [11] because clinical data managements are very heterogeneous, and the open source solutions do not play a major role in clinical trial data management [12]. However, because of flexibility, increased innovation, shorter development times, and faster procurement processes, open source software may be attracted by an organization. Also by using open source software, an organization will not be locked into using a proprietary software program. One disadvantage in an open source solution is that, due to the web-based nature of this system, it may pose a challenge for real time data entry because in many developing country settings internet connectivity is a problem. One may also face sluggish response times and system timeouts. Additionally, XML rules may adversely affect the application’s response time and therefore other options may need to be explored and used while using open software solution [3].

## Conclusion

Clinical data management has evolved and will continue to do so in response to need [13]. Limited literature hinders the capacity of scientists to design and develop a well-managed data system for their studies [14]. An ill-managed data system may lead to false outcomes, which is detrimental for the study; eventually for human health. The concepts and ideas we discussed in this paper may be useful for designing and developing a well-managed data system for the clinical studies. By controlling both the data and field activities in a system, the investigators may overcome the complexities of the visit schedules in their studies. We believe such a system would be useful for the investigators who want to initiate a complex clinical study.

## Availability of supporting data

This paper describes the design, development, and implementation of a data system. The source codes of the data system and operators’ manual can be made available upon request.

## Abbreviations

GCP: good clinical practice
TE: tropical enteropathy
IPV: inactivated polio vaccine
OPV: oral polio vaccine
EPI: expanded program on immunization
ID: identification
WHO: World Health Organization
SD: standard deviation
